# An overview of carbonic anhydrases and membrane channels of synoviocytes in inflamed joints

**DOI:** 10.1080/14756366.2019.1659791

**Published:** 2019-09-03

**Authors:** Min Jeong Ji, Jeong Hee Hong

**Affiliations:** Department of Physiology, College of Medicine, Gachon University, Lee Gil Ya Cancer and Diabetes Institute, Incheon, South Korea

**Keywords:** Synoviocytes, migration, carbonic anhydrases, aquaporins, ion channels

## Abstract

The highly aggressive fibroblast-like synoviocytes (FLSs) are inflammatory mediators involved in synovial joint destruction. Membrane channels and transporters are essential components of the cell migration apparatus and are involved in various cellular functions. Although evidence is emerging that cell migration is a physiological/pathological process, the mechanism of highly dynamic synoviocytes linked to the membrane channels and carbonic anhydrases (CAs) in inflamed joints is only partially understood. In this review, topics covered will give a brief overview of CAs and the membrane channels of synoviocytes. We have also systematically focused on the role of FLS channels and transporters under various conditions, including rheumatoid arthritis (RA), to understand the pathophysiology of the migration of synoviocytes as inflammatory mediators in joints.

## 1. Introduction

**Figure 1.**
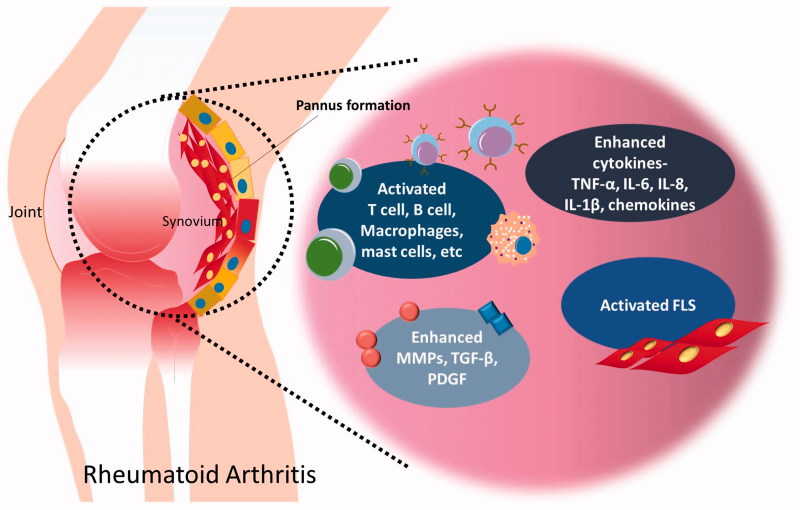
Inflammatory mediators in pathogenesis of RA. Synovial fluid interacts with inflammatory cytokines and immune cells, which have role in inflammation and joint destruction in RA synovium. Inflammation and hyperplasia of FLS involve in pannus formation of joint.

Rheumatoid arthritis (RA) is a common inflammatory autoimmune disease that induces diarthrodial joint inflammation^1^. The fibroblast-like synoviocytes (FLSs), located in the synovium, mediate synovial joint destruction by releasing metalloproteinases (MMPs) and secreting cytokines, including interleukin (IL)-6, IL-Iβ, IL-8, and tumour necrosis factor (TNF)-α in RA^2–6^. Immune cells including macrophages, T cells, B cells, mast cells, and etc. are activated in RA and play crucial roles to secrete various cytokines and mediate inflammation of joint^7^. Moreover, TGF-β and platelet-derived growth factor (PDGF) levels were elevated in the RA synovial fluids^8^^,^^9^. The inflamed synovium activates local FLS and induces the invasion of FLS^10^. Figure 1 represents the inflammatory mediators including immune cells and cytokines in pathogenesis of RA.

Cellular migration is not only an important physiological process related to wound healing, immune defence, and angiogenesis but also affects pathological processes, including tumour metastases and arthritis^11^. Moreover, the maintenance of a polarised state is the basis for cell migration^12^. Although the morphological polarisation of neuronal cells and epithelial cells is more critical than that of migrating cells, several mechanisms of the polarisation among classical polarised cells, such as neuronal and epithelial cells, and migrating cells follow similar principles. FLS are highly dynamic, and the chemotactic ability of the inflamed synovial fluid provides direction to the migration, invasion, and inflammatory/proliferative signalling events of pannus formation in the joint. To our knowledge, the membrane channels of FLS in migration have not been elucidated systematically. There is potential evidence for the involvement of carbonic anhydrases (CAs) and membrane channels in FLS migration in inflamed joints and has been discussed in the following sections.

## 2. Features of FLS

Normal synovial tissue is divided into two functional layers: a surface layer and sublining layer^13^. The surface layer is in direct contact with the intra-articular cavity, 1–2 layers deep consisting of two predominating cell types: macrophage-like synoviocytes (type A, MLS) derived from bone marrow, and FLS descended from the mesenchymal stem cells (type B)^10^^,^^13^. Those two cell types are essential for maintaining joint homeostasis. The FLS is relatively richer than MLS and displays a variety of surface adhesion molecules, such as ICAM-1, CD90, and matrix proteins to help in the anchoring with extracellular matrix^14^^,^^15^. The FLS contains endocrine and sensory functions and also possesses the epithelium-like nature^16^. While the typical feature of FLS is synovial hyperplasia during the inflammation, this review is designed to understand the dynamic role of FLS in the pathogenesis of RA and its associated membrane channels.

## 3. Carbonic anhydrases

Carbonic anhydrases are zinc metalloenzymes. The physiological role of CAs is related to membrane transporters and will be discussed in brief. CAs catalyse the reversible reactions of CO_2_ and water to produce H^+^ and HCO_3_^–^. They play a prominent role in the transport of CO_2_ and protons across biological membranes, such as intercellular, intracellular, and extracellular spaces and are involved in diverse physiological functions, including pH regulation, fluids, and enzymes secretion and bone resorption^17–19^. Several CA isozymes are expressed in mammals^20^. Architecturally, CAs include cytosolic isoenzymes (CA I, CA II, CA III, CA VII, and CA VIII), membrane-associated isozymes (CA IV, CA IX, CA XII, and CA XIV), mitochondrial isoenzymes (CA VA and CA VB), and secreted CA isoenzyme (CA VI)^21^. CAs produce HCO_3_^–^, which fuels the HCO_3_^–^ transporter^22^. The Na^+^/HCO_3_^–^ cotransporter, NBC1 cooperates with CA II and CA IV to modulate the intracellular pH^23^. High H^+^ concentration is found in the synovial fluid of RA patients, indicating that acidic pH reflects the pathophysiology of inflammation^24^. CA and HCO_3_^–^-modulating transporters contribute to the modulation of synovial pH. The intensity of the inflammation process and ache-related symptoms in RA-affected patients inversely correlate with the tissue pH values^24^^,^^25^. Tissue acidosis was found to be unfavourable for the progression of both antibody-mediated and cellular immunity processes^26^. Although evidence indicates the importance of pH regulation of synovial tissues in various conditions, the precise role of the pH regulatory mechanism and its related transporters needs to be clarified. Beyond pH regulation, substantial evidence has indicated that CAs are also involved in bone resorption, hypoxia, and autoantibody formation. Expression of CA I was increased in the synovium of spondylitis and may accelerate calcification and bone resorption^27^. The overexpression of CA IX and XII, associated with tumour hypoxia, was revealed in the inflamed synovium^28^^,^^29^. Enhanced oxidative stress of erythrocytes in RA has addressed its correlation with CA II autoantibody formation^30^.

Despite these differences in the role of CA, the regulatory role of CA in transporter machinery involves cell migration coordinated with cell adhesion molecules and ion transporters. The function of the CAs was to acidify the extracellular environment, thereby reducing cell adhesion and consequently increasing invasion and migration of tumour cells^31^. Especially, CA IX and CA XII were enhanced by hypoxic condition in tumour cells^32^. Hypoxia-inducible factor (HIF) affected the migration, cellular pH, and cell survival associated with tumour growth^33^. The CA IX has been linked to cell–cell connections in the cell membrane, controlled by E-cadherin^34^. It also regulated the cell migration by inhibiting E-cadherin associated with cell adhesion and interacting with the bicarbonate transporter, anion exchanger 2, in the leading edge regions in SiHa cells^31^. Bicarbonate transporters not only controlled the pH of the cells but also affected cell migration^35^. The deficiency of SLC4A4 (NBCe1), an electrogenic Na^+^/HCO_3_^–^ co-transporter, was influenced by cell migration by interfering with the intracellular pH regulatory mechanism in MDA-MB 231 breast cancer cells^36^. However, information on CAs on the RA FLS remains unclear. The verification of regulatory and migration role of CAs in FLS will provide a new scope for synovial physiology.

## 4. Membrane ion channels of FLS

### 4.1. Aquaporins

The aquaporins (AQPs) are water or small molecule-transporting channel proteins across the plasma membranes of various human tissues and cell types^37^. Thirteen types of AQPs (AQP0–AQP12) from mammalian tissues have been cloned and sequenced^38^. The AQPs are classified into two groups: water selective channel (orthodox AQPs) and water, glycerol, nitrate (AQP6), and urea channel (aquaglyceroporins; AQP 3, AQP7, and AQP9)^39^. The permeability of AQPs is dependent on osmotic and hydrostatic gradients and pH values. Several investigations have shown the involvement of AQPs in cartilage damage in joint diseases like RA and osteoarthritis (OA). AQP1 is distributed in the articular cartilage and the synovium^40^. AQP1 is also expressed in chondrocytes and synoviocytes of RA patients^41^. Up-regulated AQP1 found in the inflamed synovial tissues of RA patients might play a potential pathological role in hydrarthrosis and joint swelling^42^. Acetazolamide, AQP1 inhibitor, was decreased AQP1 protein level via inhibition of NF-κB activation and subsequent reduction of hind pow swelling in adjuvant-induced arthritis rats, suggesting that attenuation of AQP1 mediates anti-arthritis effect^42^. It is well-known that AQP4 possesses high water permeability than that of AQP1^43^ and its role in the nervous system has been studied^44^. AQP4 is over-activated in rat articular chondrocytes and high homologues of AQP4 between rat and human^45^; however, the pathological role of AQP4 in RA is still unclear. AQP9 was strongly induced upon treatment with TNF-α in FLS and was also expressed in the RA and OA synovial tissues^41^. Although the pathological roles of AQP in the synovial tissues remain to be elucidated, experimental evidence has revealed that AQPs are involved in the pathogenesis of hydrarthrosis and synovitis (Table 1).

**Table 1.** AQPs in FLS.

AQP	Mechanism	Species	Ref.
AQP1	Hydrarthrosis and joint swelling Inhibiting NF-κB pathway by AQP1 inhibitor	Adjuvant-injected arthritis rats	^42^
AQP4	Over-activated AQP4 in articular chondrocytes	Articular chondrocytes, adjuvant-injected arthritis rats	^45^
AQP9	Hydrarthrosis	HepG2, FLS from OA and RA patients	^41^

**Table 2.** TRP channels in FLS.

TRPs	Mechanism	Species	Ref.
TRPC1/TRPC5	Reduced MMP secretion and joint inflammation	Human FLS, mouse joint tissue	^76^^,^^77^
TRPV1	Promoted inflammation and joint destruction	SW982 human synovial cells	^88^
TRPV2	Reduced expression of the MMP2 and MMP3 proteins	FLS from DA (severe and erosive arthritis)	^90^
TRPV4	Reduced IL-8 production	FLS with RA and without RA, MH7A	^89^
TRPA1	Increased pain-related response	Human FLS, ddY mice	^75^
TRPM3	Decreased hyaluronan secretion	HIG-82 cells (FLS cell-line), joint tissue of RA patients	^94^
TRPM7	Activated ER stress, increased apoptosis of FLS	FLS from RA	^99^

### 4.2. K^+^ channels

Ca^2+^-activated potassium channel K_Ca_1.1 (known as BK, Maxi-K, Slo1, or *KCNMA1*) is the only member of the K_Ca_1.1 potassium channel family^46^. The K_Ca_1.1 channel consists of α-subunits and β-subunits comprising of four different isoforms (β_1_, β_2_, β_3_, and β_4_)^47^. The K_Ca_1.1 was a major K^+^ channel expressed in FLS plasma membrane in RA^48^. Blocking the K_Ca_1.1 channel in RA FLS by inhibiting the α-subunit interrupted Ca^2+^ homeostasis; the proliferation, migration, and the invasiveness of cells; and the cytokines and chemokines^48^. The K^+^ channels in the plasma membrane of cells play a critical role in regulating β_1_ integrins by influencing Ca^2+^ homeostasis^49^. The FLS cells express a variety of integrins, α_4_, α_5_, α_6_, and β_1_ isotype^50^. Blocking of K_Ca_1.1 channel interrupted Ca^2+^ homeostasis, thus affecting integrin expression^49^. Enhanced integrin ligation increased cytokine signalling and growth factor expression, thus leading to the expression of matrix MMPs^50^. Blocking of K_Ca_1.1 activity or its expression reduced the FLS proliferation and expression of pro-MMP2 and attenuated the subsequent FLS invasion. On the contrary, activated K_Ca_1.1 or overexpression of the channel enhanced the invasiveness of FLS^51^. Regulation of K_Ca_1.1 of FLS also affected the proliferation and migration of CCR7^–^ effector memory T cells, another major cell type implicated in the progression of RA^52^.

### 4.3. Acid-sensing ion channels

Acid-sensing ion channels (ASICs) mediate tissue acidosis by pH changes are known as voltage-insensitive, ligand-gated cation channels with protons^53^^,^^54^. The ASICs are associated with inflammatory pain, and especially ASIC1 and ASIC3 contribute to the musculoskeletal pain^55^. The ASIC3 is expressed in the sensory neurons that innervate the synovial joints by increasing the intracellular Ca^2+^ levels upon sensing a decrease of pH in the inflamed joint^56^^,^^57^. Synovial inflammation and inflammatory cytokine levels were increased that led to joint destruction in ASIC3–/– mice^55^. FLS were activated with the decrease in pH; the acidic environment increased the intracellular Ca^2+^ levels by ASIC3^57^. Activation of FLS in acidic pH mediates the accumulation of inflammatory cytokines. In addition, activation of ASIC3 by acidic pH evokes Ca^2+^ signalling, which lead to the apoptosis of FLS by phosphorylation of the MAP kinase ERK in synovial inflammation; thus, it could be a blockade of synovial proliferation^58^. Activation of ASIC3 can be a therapeutic strategy for reducing inflammatory FLS level and subsequent disease progression in an inflamed joint.

### 4.4. Ca^2+^ signalling of FLS

Intracellular Ca^2+^ plays crucial roles in various physiological processes, including the flow of nerve impulses, muscle contraction, cell division, and hormone secretion^59^. Enhanced Ca^2+^-activated phosphatase calcineurin activity and Ca^2+^ release by proinflammatory cytokine were observed in RA FLS, suggesting that dysregulated Ca^2+^ signalling involved in the pathogenesis of chronic arthritis^60^. In addition, synovial fluid of patients with RA contains ATP^61^ and FLS expressed P2X7 receptor and functionally involved in ATP-dependent Ca^2+^ release and subsequently mediated IL-6 release^62^. Generally, the cytosol is surrounded by two major Ca^2+^ sources; the intracellular Ca^2+^ stores including sarco/endoplasmic reticulum (SR/ER), nucleus, golgi, and mitochondria and the extracellular media^63^. The Ca^2+^ is released from intracellular stores or enters into the cells through the plasma membrane^64^. The Ca^2+^ homeostasis is maintained by two types of membrane ATPase, the SR/ER Ca^2+^-ATPase (SERCA) and plasma membrane Ca^2+^-ATPase (PMCA). These pumps are involved in reduction of cytosolic Ca^2+^, from cytosol to intracellular Ca^2+^ stores by the SERCA and to the extracellular space by the PMCA^65^. Na^+^/Ca^2+^ exchangers are also known to have a critical role in Ca^2+^ removing mechanism with Na^+^ regulation^66^^,^^67^. The ER also contains inositol-1,4,5-trisphosphate (IP_3_) receptors (IP_3_Rs) and ryanodine receptors (RyRs), which provide conduits for the rapid release of Ca^2+^^68^. The agonist stimulation such as receptor activation leads to the generation of IP_3_, which releases to the cytosol and binds to the intracellular membranes to release Ca^2+^ from the intracellular stores of Ca^2+^^69^. Although the Ca^2+^ signalling and its signalling proteins have been well established, the network of Ca^2+^ signalling in FLS needs to be clarified more extensively.

### 4.5. TRP channels

**Figure 2.**
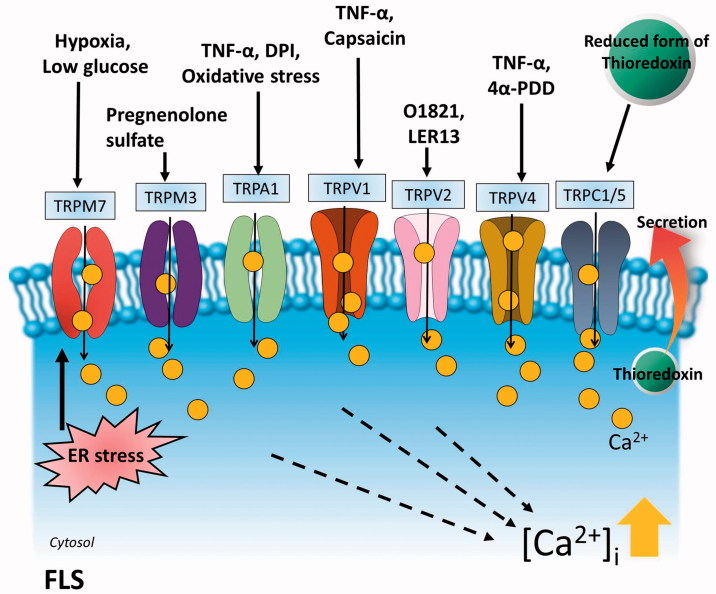
Activators of TRP channels in RA FLS. The activators of FLS-associated TRP channels are summarised. The mechanism of action is represented in Table 2. Activation of TRP channels increases intracellular Ca^2+^ level and is involved in various functions including the reduced MMP secretion, joint destruction, enhancement of pain, and apoptosis of inflamed FLS. TRPV: transient receptor potential vanilloid channels; TRPC: transient receptor potential canonical channels; TRPM: transient receptor potential melastatin channels; TRPA: transient receptor potential ankyrin channels.

Although there is relatively low evidence in Ca^2+^ signalling network in FLS, studies of transient receptor potential (TRP) channels have been performed in various reports. The TRP channels have been known to be nonselective cation channels and play a critical role in inflammatory pain of arthritis^70^^,^^71^. FLS express the TRP family proteins, including TRPC (TRPC-canonical) 1, TRPC5, TRPA (TRP-ankyrin) 1, TRPV (TRPV-vanilloid) 1, TRPV2, TRPV4, TRPM (TRPM-melastatin) 7, and TRPM8^72–76^. We will discuss FLS-related TRP channel activation and will provide information on the following section. The detailed mechanism is summarised in Figure 2 and Table 2.

#### 4.5.1. TRPC

The TRPC1 and TRPC5 were expressed in secretory FLS^76^^,^^77^. The expression of oxidoreductase thioredoxin, a well-known oxidative stress marker, was increased in RA synovial fluids to counteract oxidative stress^78–80^. The thioredoxin was considered as a costimulatory component with cytokines in FLS^81^ and can be secreted^82^. Extracellular reduced form of thioredoxin enhanced the activities of TRPC1 and TRPC5 channels as new extracellular targets^77^. Inhibition of these channels by antibodies enhanced MMP secretion and suppressed the thioredoxin-mediated inhibitory effect on secretion^77^. More recently, the study of TRPC5 KO mice and inhibition of TRPC5 channels by antagonist addressed the enhanced inflamed joint and hyperalgesia^76^, suggesting that functional modulation of TRPC 1/5 could be considered as therapeutic targets for RA.

#### 4.5.2. TRPA

The TRPA1 is a cold-sensitive and Ca^2+^-permeable nonselective cation channel and plays an essential role in inflammation and pain^83^. For the evidence of TRPA1 expression in FLS, mRNA of TRPA1 has been detected in SW982 human synoviocytes^74^. Diphenyleneiodonium (DPI) as a TRPA1 activator induced Ca^2+^ signal in TRPA1-expressing FLS and pain response in ddY mice^75^. More recently, it has been reported that proinflammatory FLS can be attenuated by TRPA1 activation. TNF-stimulated FLS enhanced protein level of TRPA1 and subsequent stimulation of TRPA1 enhanced the necrosis^84^.

#### 4.5.3. TRPV

The TRPV channels sense heat, protons, lipids, and osmolarity^85^^,^^86^. The RA and OA patients possess pain linked to TRPV1^73^. Capsaicin, an agonist of TRPV1, increased IL-6 mRNA and protein levels by promoting pro-inflammatory cytokines^87^. Activation of TRPV1 enhanced mRNA level and protein level of IL-6 in FLS from RA and OA patient and application of TRPV1 antagonist could be therapeutic strategy to modulate nociception from arthritis^73^. Activated FLS-mediated TNF-α secretion enhanced the expression of TRPV1 and TRPV4 in SW982 human synoviocytes^88^. Especially, 4α-phorbol-12,13-didecanoate (4α-PDD), a selective TRPV4 agonist and hypotonic stimulation induced an increased intracellular Ca^2+^ level and decreased IL-8 secretion in RA^89^. The enhanced TRPV2 expression was associated with invasion of FLS from rats using gene profile technique^72^. However, functional activation of TRPV2 by specific TRPV2 agonists, O1821 and LER13, dramatically reduced IL-1β-mediated expression of the MMP2 and MMP3 proteins in FLS and reduced the severity of disease and genetic deletion of TRPV2 enhanced the invasiveness of FLS^90^. Expression of TRPV2 involves in invasion mechanism and further functional stimulation of TRPV2 attenuates the invasiveness, suggesting that regulation of TRPV2 can be also novel therapeutic strategy of RA such as TRPC and TRPA channels.

#### 4.5.4. TRPM

The Ca^2+^ entry through TRPM3 is involved in cell survival, death, growth, and differentiation^91^. Hyaluronan, known as the major component of the extracellular matrix, was increased in RA patients^92^. The increased secretion of hyaluronan from RA FLS was reduced by TRPM3 activator pregnenolone sulphate, activating TRPM3-mediated Ca^2+^ entry^93^^,^^94^. The TRPM7 mediates a variety of functions, such as cell cycle, migration differentiation, and regulation of Ca^2+^ homeostasis and it is correlated with the oxidative stress-induced cell injury^95–97^. It has been proposed that hypoxia and low glucose also lead to ER stress in RA joints^98^. Inhibition of TRPM7 by Gd^3+^ and 2-aminoethoxydiphenyl borate (2-APB) induced RA FLS apoptosis by activating ER stress^99^.

## 5. Future perspectives

**Figure 3.**
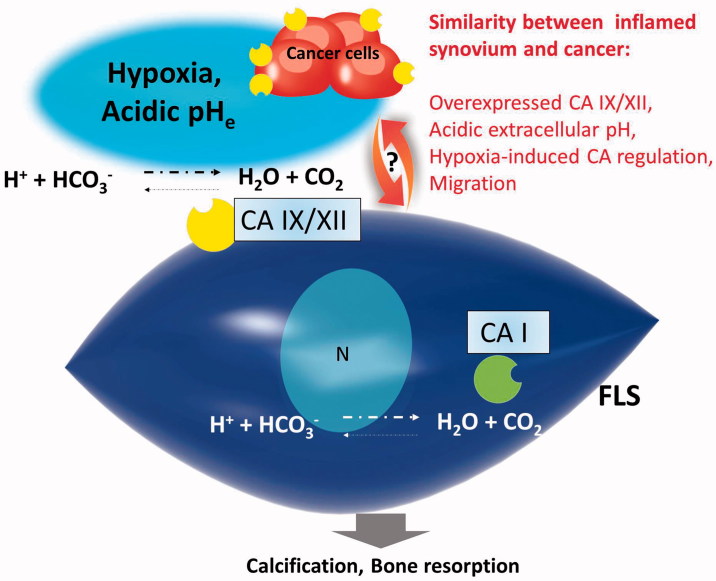
Potential function of CAs in inflamed synovium. CA I was overexpressed in the synovium of the patients with ankylosing spondylitis^27^. The overexpression of CA IX and XII was revealed in the inflamed synovium. Although determination of CA isoenzymes on the RA FLS remains unclear, there are several similarities between inflamed synovium and cancer. CA: carbonic anhydrase; pH_e_: extracellular pH.

We limited our review to the most relevant channels related to cell migration in RA FLS. Evidence related to cell migration by water and ion channels addressed the housekeeping functions. The migration or invasion is the major feature of cancer cell. The major consequences between the cancer cells and inflamed FLS are hypoxia and acidic circumstances^100^. The cancer cells employ a circuit of ion transporters and enzymes to avoid the detrimental consequences of hypoxic and acidic tumour microenvironment. Alterations of CA IX and CA XII are associated with various cancers and considered oncogenic factors^101^. The overexpression of CA IX and XII in the inflamed synovium^28^^,^^29^ provides the several similarities of pathology between inflamed synovium and cancer (Figure 3). Such similarities between microenvironments including hypoxia, acidic pH, and enhanced CA IX and XII can be speculated, as RA FLS would share the migration mechanism with cancer cells. Thus, therapeutic options in cancer therapy can be expanded and exploited for the RA model.

Currently, experimental evidence for the involvement of CAs and FLS membrane channels in RA is limited. The physiological and pathological roles of ion channels and transporters in dynamic FLS migration have not yet been studied in detail. Here, we have summarised the studies on membrane channels and regulatory enzymes of RA-FLS with an aim to understand their migrated state. However, many questions regarding RA-FLS still need to be clarified. What are the exact molecular mechanisms by which ion transporter affects the FLS migration apparatus? What are the exact components of synovial fluid that mediate the FLS dynamics? What are the components affecting the differential expression of CAs and membrane channels in FLS? What is the combined mechanism of CAs as regulatory enzymes? Several membrane channels and transporters show tissue-specific expression. Thus, unravelling the mechanisms by which ion channels and transporters are positioned in and modulate the migration of activated FLS will be a rewarding pursuit for the coming years. The motivation of channel physiologists is also needed to develop potential therapeutics to counter the critical pathophysiological involvement of FLS migration in joints in RA.
